# Potential for Further Mismanagement of Fever During COVID-19 Pandemic: Possible Causes and Impacts

**DOI:** 10.3389/fmed.2022.751929

**Published:** 2022-03-02

**Authors:** Samer Singh, Dhiraj Kishore, Rakesh K. Singh

**Affiliations:** ^1^Centre of Experimental Medicine and Surgery, Institute of Medical Sciences, Banaras Hindu University, Varanasi, India; ^2^Department of General Medicine, Institute of Medical Sciences, Banaras Hindu University, Varanasi, India; ^3^Department of Biochemistry, Institute of Science, Banaras Hindu University, Varanasi, India

**Keywords:** benefits of fever during infection, fever management guidelines, mortality, heat shock, inflammation, antipyresis, COVID-19, respiratory diseases

## Abstract

Fever remains an integral part of acute infectious diseases management, especially for those without effective therapeutics, but the widespread myths about “fevers” and the presence of confusing guidelines from different agencies, which have heightened during the coronavirus disease 2019 (COVID-19) pandemic and are open to alternate interpretation, could deny whole populations the benefits of fever. Guidelines suggesting antipyresis for 37.8–39°C fever are concerning as 39°C boosts the protective heat-shock and immune response (humoral, cell-mediated, and nutritional) whereas ≥40°C initiates/enhances the antiviral responses and restricts high-temperature adapted pathogens, e.g., severe acute respiratory syndrome coronavirus 2 (SARS-CoV-2), strains of influenza, and measles. Urgent attention is accordingly needed to address the situation because of the potential public health consequences of the existence of conflicting guidelines in the public domain. We have in this article attempted to restate the benefits of fever in disease resolution, dispel myths, and underline the need for alignment of national treatment guidelines with that of the WHO, to promote appropriate practices and reduce the morbidity and mortality from infectious diseases, such as COVID-19.

## Introduction

Fever is integral to our natural defense against acute clinical infections, especially those without effective therapeutics, e.g., common cold, measles, and influenza (1–9). It is an evolutionary conserved adaptive response, at least >400 million years old, protecting us from potentially dangerous pathogens (1, 2, 5–7, 10–12). Kindly note, the term “fever(s)” in the manuscript narrowly applies to the elevation of body temperature in response to an infection, unlike its broader usage in the literature for any temperature elevation. The beneficial role of fever in controlling infectious disease is well recognized and remains part of standard immunology textbooks. *Janeway's Immunobiology* states “At higher temperatures, bacterial and viral replication is less efficient, whereas the adaptive immune response operates more efficiently” [(13), p.110]. The *Kuby Immunology* (14) describes “fevers” as “helps to eliminate many temperature-sensitive bacterial strains” (p. 223), “a protective response, as elevated body temperature inhibits replication of some pathogens” (p.323), “decrease microbial viability” (p. 401), etc. *Saladin's Anatomy and Physiology*, summarizes its role as “Fever is beneficial in that it (1) promotes interferon activity, (2) inhibits the reproduction of bacteria and viruses, and (3) elevates metabolic rate and accelerates tissue repair.” [(15), p.818]. However, the value of fever (≥39°C) in infectious disease resolution and control remains highly underappreciated in the age of antimicrobials and vaccines. The practice of antipyresis has changed tremendously in the last 50–60 years (16–25). It is being recommended for temperatures as low as 37.8–38°C (26, 27).

Recent publications of advisories and guidelines for coronavirus disease 2019 (COVID-19) management (28–31), in disagreement with the existing fever management guidelines from the WHO and various national/local guidelines (32–36) are concerning. Furthermore, there had been a dearth of publications on fever management during the COVID-19 pandemic. As of November 2, 2021, the “PubMed” database search for keyword “COVID-19” fetched 183,837 articles whereas keyword combinations, “COVID-19” AND “Fever”, “COVID-19” AND “Antipyresis”, “COVID-19” AND “Fever” AND “Antipyresis”, and “COVID-19” AND “Fever management” yielded 5,941, 1, 0, and 5 articles, respectively. Out of five articles on COVID-19 fever management, only one (editorial) stressed the need for personalized fever management (37). It prudently suggests ‘targeted temperature management' for patients with low O_2_ saturation or other life-threatening complications only on the need basis. Retrospective data analysis indicates that fever may increase survival in different COVID-19 situations (38). However, conflicting fever management guidelines approving aggressive antipyresis remain in the public domain (28–31) subjecting public health to infectious diseases, such as COVID-19.

The value of fever has remained well recognized through the ages in different classical treatment systems since the times of ancient physicians, such as *Charak and Hippocrates* (39–42). The Ayurveda classics, such as “*Charak* (also *Charaka* or *Caraka) Samhita*” (39) and the current naturopathy practice, recognize the role of fever as a facilitator of the elimination of pathogens or toxins (“*doshas*”) from the body. In modern times, the early 20th-century work of Julius Wagner-Jauregg on neurosyphilis or “dementia paralytica”—an untreatable terminal manifestation of syphilis characterized by progressive paralysis and insanity (43), reestablished and popularized the therapeutic potential of fever. His experimentation with different fever-inducing agents, e.g., tuberculin, streptococci, and malaria parasites, identified malarial parasite inoculation as the most satisfactory method for treatment (44). For this monumental work, he received the Nobel Prize in 1927. Wagner hypothesized the curative potential of fever based upon the reduced occurrence of “dementia paralytica” in malaria-endemic regions and the reported spontaneous curing in isolated cases after a febrile illness (45). Analogous reduced COVID-19 mortality has been observed in malaria and tuberculosis endemic regions of the world (46, 47). The populations' genetic constitution and the cellular immunity-boosting effect of these infections have been proposed as protective determinants (46, 48). The reduced likelihood of aggressive antipyresis practice in these poor regions could have been another favorable determinant as temperatures ≥39°C inhibited severe acute respiratory syndrome coronavirus 2 (SARS-CoV-2) replication (49).

We have attempted in this manuscript to further restate the important role of fever in infectious disease resolution, and dispel the basis for fever paranoia. In addition, we discuss the need to harmonize the many publicly available guidelines on fever management with that of the WHO, particularly concerning the management of COVID-19.

### Role of Fever in Response to Infectious Diseases

Fever plays multiple roles in infectious disease control (1–25). During acute infections, the brain-controlled incremental elevation of body temperature up to 39–42°C is seen over several days with intervening normothermia (no fever). Literally, each temperature elevation increases the body's thermotolerance—preparing it for the next higher temperature exposure possibly required to stop the pathogen replication and further enhance the immune response/functions.

Fever of about 39°C boosts the production and accumulation of various heat shock proteins (HSPs) and metallothioneins as a part of protective heat shock response (HSR) to prepare the host cells against any potentially damaging higher or ‘supraphysiological temperatures' exposure and inflammatory response (50–54). It helps amicably resolve any threatening stressful situations of thermal, oxidative, and metabolic nature arising from an overwhelming inflammatory response to the persisting infection (55–57). The repeated heat shocks (such as, recurrent fever), estrogens (E2), and cyclopentenone prostaglandins produced in later stages of disease/inflammation enhance HSR. Mechanistic insights about the potential protective role of HSR in COVID-19 have been reviewed recently (58). Incidentally, robust HSR generation is suppressed in most of the COVID-19 comorbid conditions, e.g., cardiovascular diseases, obesity, frailty, diabetes, and metabolic syndromes (58). Protective alterations in essential metal ions levels are made in different internal milieus (nutritional immunity) during the acute-phase response (59–62). For example, reduced serum zinc levels protect the host tissues from oxidative damage and enhance the chemotaxis and immune cells' activity, such as the targeted intracellular pathogen killing (63–65). Zinc supplementation during infections has been correlated with increased mortality in diseases, such as HIV and COVID-19 (66, 67).

Temperature elevation to about 40°C enhances the production of antiviral interferons (IFNs) (~10 times) and various interleukins that promote pathogen clearance and reduce inflammatory damage (1, 54, 68–70). The IFNs help the surrounding uninfected cells attain a viral infection refractory state through the induction of a complex web of host genes that prevent viral infection and replication (68). The IFNs mediated activation of NK cells and macrophages plays a key role in disabling the viral replication cycle by actively seeking and destroying the virus-infected cells (1, 54, 69). Additionally, it dampens the viral infection by inducing innate and intrinsic antiviral responses (71). The HSR and IFNs, together, orchestra a balanced inflammatory response that minimizes the host damage without jeopardizing an aggressive cell-mediated response for pathogen clearance, e.g., activation of cytotoxic activity of NK cells, K cells, T cells, the cytocidal activity of macrophages, and activation of suppressor T cells (1, 13, 14, 52, 54).

The appearance of increasing magnitude fevers over time, up to 42°C, is commonplace (22, 23, 72). Gradual elevation of body temperature acts as a failsafe mechanism to eliminate those pathogens that have survived/escaped (mutants) lower temperature exposures (73–76). The temperature of ≥ 39°C, suggested for antipyresis by the WHO, is “restrictive,” i.e., inhibits replication/growth, for most pathogens (73). Higher body temperatures facilitate the clearance of pathogens with higher “restrictive temperature,” e.g., 39–40°C reduces SARS-CoV-2's replication by >100-fold (49); 41–42°C reduces poliovirus' replication by ~200-fold (75) while greatly enhances the serum-induced lysis of Gram-negative bacteria (77). Temperature elevation between 35°C and 41.5°C also increases antimicrobials' efficacy by 4–16 times (74) making the treatment of infections/coinfections of inherently lower temperature niches easier (76).

The fever-induced loss of appetite (anorexia) favorably affects infectious disease resolution. The induced processes, such as unfolded protein response, classical starvation regulated responses, and ketogenesis, supposedly augment survival by reducing reactive oxygen species (ROS) mediated neural tissues damage and the inflammatory damage of other organs, such as the cardiovascular system (78–80). The anorexia-associated beneficial changes in physiology speed-up the repair and recovery process without jeopardizing immune system functions. Animal experiments indicate “fasting” in bacterial diseases and “caloric restriction” (light sugar/glucose intake) in viral disease to greatly diminish mortality rates, essentially supporting the classical notion of “feeding the flu (viral) while starving the fever (bacterial)” (81). Though there remains a dearth of human studies, the classical Indian medicine system and Ayurveda treatises, which are some of the oldest existing medical texts and form the basis of treatment (including the home remedies) for the majority in the Indian subcontinent, do recommend oddly similar personalized diet regimens for patients. For example, “*Charak Samhita”* recommends elaborate personalized feeding strategies that range from levels of fasting to light intake of sugary foods and meat-soups in respiratory tract infections for different durations as per the stage of infection and patient's condition [(39), p. 65; *Sukt*:139–141, p. 67; *Sukt*:149–156], while categorically restricts the intake of fatty and heavy food [(39), p. 80; *Sukt*:272–283]. Moreover, for acute infections, it advocates a delay in recuperative and fever-pacifying interventions till the fever matures (i.e., performed its function; up to 6 days) on the concerns of complicating the recovery [(39), p. 67; *Sukt*:160 and others], something aligned with modern understanding of the role of fever in disease resolution and the concepts of nutritional immunity (54, 59, 60). A delay of up to 10 days is suggested for weak patients who still have or had a low-grade fever [(39), p. 80; *Sukt*:272–283]. Such traditional medicine recommendations could be worth testing in different disease models as per the modern concepts to verify their applicability in infectious disease management. Finally, the benefit of fever-induced anorexia and myalgia-precipitated inactivity in reducing host tissue damage and pathogen dissemination (keeping the R_o_ down) cannot be overlooked.

### Fever Phobia: Prevalence, Possible Causes, and Implications

The phobia of fever remains extensive among the general public, nursing staff, and clinicians, despite the body of theoretical and practical/experimental evidence to the contrary (14–22, 36, 72, 82). It has been gradually increasing over the years and promoting the continuation of non-evidence-based irrational management practices (16–25, 72, 82). A rapid increase in unawareness of the role of fever in infections and fever phobia was observed from the 1980s to 2010 among clinicians (12–65%) (3, 22, 23), nursing staff (70%) (83, 84) and parents (60% to 90–95%) in the United States (3, 19, 22–25, 83) and other countries (21, 24, 25, 42). Considering the potential impact of unnecessary antipyretics usage on public health (3, 5–7, 11, 12, 70), the WHO and various national agencies have made efforts to increase the awareness among the stakeholders to improve fever management practices (32–36).

Some potential underlying causes promoting the current notion of treating every “fever” along with possible implications are briefly discussed below:

1. *Inability to differentiate fever by type and terminology mess*: The inability to differentiate the common “hyperpyrexia” from “hyperthermia” is widespread. The former beneficial one results from the brain-controlled incremental increase in temperature during infection, while the latter concerning one results from uncontrolled temperature elevation on the failure of thermoregulatory mechanism as observed in heat shock (15). The pervasive confusion partly stems from the liberal usage of terms “fever,” “pyrexia,” and “Hyperthermia” in literature for a range of temperatures above 37°C. In the literature, the term “hyperpyrexia” has been used for anesthesia-driven uncontrolled temperature elevation as well (85). It makes the situation chaotic, as any temperature elevation could be construed as “hyperthermia” so naturally “dangerous” and “fit for treatment”. The coining and consistent usage of the precise terms for different fever types is required. Reserving the usage of “hyperpyrexia” or more explanatory term “pathogen-hyperpyrexia” for the fevers of infectious disease origin while “hyperthermia” for non-brain-controlled fever is suggested to reduce the prevalent confusion among clinicians and public concerning their management. In clinical practice, many a time it is difficult to know the origin of a fever, so the alleviation of symptoms takes precedence as “why take chances” when antimicrobials and antipyretics are readily available (86, 87). However, the prescription of antipyretics for diseases without effective therapeutics makes it a risky proposition (e.g., COVID-19, influenzae, and measles).
2. *Fear of Brain Damage From High Fever:* The widely ingrained fear of brain damage including among physicians perceptibly originates from the experience of seizures and writhing observed around 39°C in 2–5% children <5-year-old (23, 32, 70, 72), largely ignoring the hard evidence that in almost last one century no one died or had brain damage from the normal course of fever during infections, except an often-quoted anecdotal report from the 1950s (88). Still up to 65% of physicians considered fever as harmful and 90% believed that febrile convulsions could cause brain damage (20, 22, 23) even though temperatures up to 42°C (108°F) are safe in infections [(22, 23, 72); as shown in Table 1]. Most cases of irreversible brain damage or death have occurred from fevers that reached 44–46°C (111–115°F) [(15), p. 819]. So, the fear of brain damage from 40°C fever could be valid for “hyperthermia” but unfounded for “pathogen-hyperpyrexia”.
3. *Most dangerous pathogens get restricted at 39*°*C*: Antipyresis at ≥39°C has been mostly inconsequential in clinical practice since most pathogens cannot replicate above this temperature (73). However, for infections caused by high temperature adapted (>39°C) pathogens, the effect of antipyresis on patients could range from inconsequential to disastrous depending upon their genetic makeup, the virulence of the pathogen, prevailing protective immunity/previous exposure, health status, age, comorbidities, etc. (as shown in the Section below).
4. “*No evidence of ill effects of fever reduction” in clinical practice and equating “no evidence of harm” with “evidence of no harm.”* Since the 1950s, progressively, most acute diseases have been controlled by vaccines. Availability of antibiotics/antivirals has made fever redundant for common infections. Many times, physicians make the oversight of equating “no evidence of harm” with “evidence of no harm” in prescriptions. Though largely understandable in their practice, these are fatal flaws when dealing with novel diseases. For example, seemingly “harmless” antipyresis in infections without any effective therapeutics could allow rapid pathogen growth, deny timely immune enhancement, and increase the vulnerability to adverse outcomes from future oxidative stress, inflammatory damage, etc. Additionally, normothermia promoted mobility would allow wider dissemination of pathogens as modeled for influenza (93).
5. *Need of More Context Clarifying Remarks in Medical Textbooks:* The under-appreciation of the role of fever in disease resolution and perpetuation of myths among medical students and practitioners could be also partially stemming from extant remarks present in many widely used medical textbooks that can be easily taken out of the context. For example, *Harrison's Principles of Internal Medicine*, [(94), p.104] under “The Decision To Treat Fever” states “treatment of fever and its symptoms does no harm and does not slow the resolution of common viral and bacterial infections.” The statement is perfectly all right. However, many could err to equate “common” with “all” infections in their practice where effective therapeutics may not be “commonly” available. They may miss the immediate context, i.e., “Most fevers are associated with self-limited infections, such as common viral diseases.” Generalization of something applicable to “common” and “self-limiting diseases” to “all diseases and conditions” could have fatal consequences in those rare or novel infections that lack the effective therapeutics or the therapeutics are not being co-administered. Inclusion of more context clarifying texts in the reference textbooks could help the message get better understood as fever has an unquestionably essential role in infectious disease resolution (1–9).
6. *Lack of clear guidelines and the legal framework for the protection of patients and physicians during pandemics:* Pandemics require clear, concise, evidence-based, precise guidelines along with a foolproof legal framework that ensure the protection of patients and physicians (95). During the COVID-19 pandemic, the fear of inviting the wrath of government agencies or litigation on not following the treatment guidelines (96, 97) could have significantly increased the antipyretics prescriptions even for uncertified conditions. Many times, the detection of fever would have caused treatment delays and incorrect patient management under different clinical contexts (98). The lack of universally agreed-on recommendations and legal safety/protection systems for physicians and patients would be adversely affecting the existing healthcare systems, including the increased morbidity, disability, and mortality from unrelated conditions that required direct contact, e.g., elective surgeries and preventive ocular surgeries (99–101). Despite the potential, the fear of government action on mandated treatment guidelines non-pursuance would be also adversely affecting the application of therapy options and fever management practices from established traditional medicine systems (e.g., Ayurveda, Chinese, and Korean), further aggravating the management of infectious diseases (102–104). The prospection/evaluation and potential application/repurposing of the available antiviral medications and vaccines from modern medicine could be affected (48, 105–107).

**Table 1:** Myths and facts about infection caused fever (pyrexia or pathogen-hyperpyrexia).

**S.No**.	**Myths (72)**	**Facts (72)**	**Comments**
1.	*“***fever will hurt their child**” Or them (FEVER PHOBIA)*	“*In fact, fevers are harmless and often helpful.”* [*Still, the **Most Pervasive Fear** Among **Pediatricians (65%), Nursing Staff (70%)**, and **Adults (95%) for indicating aggressive fever reduction treatment**] *Myth originated from the observations in uncontrolled fever (Thermia)	**Myth NOT** applicable to brain-controlled harmless fever “*Pyrexia*” – for infection control. **Fevers are most helpful for bacterial & viral infections without therapeutics**.
2.	***“All fevers are bad for children”***	“*Fevers turn on the body's immune system. They help the body fight infection. **Normal fevers between 100°and 104°F (37.8°–40°C) are good for sick children**.”*	***Note** that **40°C** is more than the **39°C** limit currently erroneously suggested by many for COVID-19 and other viral fever patients who are mostly adults.*
3.	**“Fevers above 104°F (40°C) are dangerous. They can cause brain damage.”**	***“Fevers with infections don't cause brain damage***. ***Only temperatures above 108°F (42°C) can cause brain damage. It's very rare for the body temperature to climb this high**. It only happens if the air temperature is very high. An example is a child left in a closed car during hot weather.”*	**Fevers don't cause brain damage**. **Fevers around 108F are a rarity but still harmless** and can be observed in hot weather or cases when infection could not get controlled at lower temperatures observed previously. **Brain damage and deaths have been reported at 44°to 46°C (111°to 115°F)** **(****15****)**
4.	***“Without treatment, fevers will keep going higher***.”	• ***“Wrong***, because the ***brain knows when the body is too hot***. • Most fevers from infection don't go above 103° or 104° F (39.5° –40°C). • They rarely go to 105° or 106° F (40.6° or 41.1°C). ***While these are “high” fevers, they also are harmless ones***.”	• In infections, the level of fever **(Pyrexia) is actively controlled** by the brain, **unlike hyperthermia**. • Generally bacterial and viral. • Mostly viral
5.	“With treatment, fevers should come down to normal.”	“With treatment, most fevers come down 2° or 3° F (1° or 1.5°C)”	**Unnecessary dosage or increase in dosage is futile for** ***Pyrexia*** **causing hepatotoxicity** **(****3**, **70**, **89****–****92****)**. Frequent dosages are needed to manage Hyperthermia.
6.	“Anyone can have a seizure triggered by fever.”	“Only 4% of children can have a seizure with fever.”	Harmless febrile seizures occur in 3–5% of genetically susceptible children (23).
7.	“Seizures with fever are harmful.”	“These seizures are scary to watch, but they stop within 5 minutes. **They don't cause any permanent harm**. They don't increase the risk for speech delays, learning problems, or seizures without fever.”	**Seizures are harmless** and don't cause any permanent brain damage.
8.	“All fevers need to be treated with fever medicine.”	***“Fevers only need to be treated if they cause discomfort*** (makes your child feel bad). Most fevers don't cause discomfort until they go above 102° or 103° F (39° or 39.5°C).”	**For adults, the threshold for discomfort is generally around 41**^**o**^**C or higher**. Fevers help early resolution of disease
9.	“The exact number of the temperature is very important.”	“How your child looks and acts is what's important. The exact number of the temperature is not.”	It applies to adults as well. If it feels very sick (discomfort), the cause is more likely to be serious & needs medical attention.
10.	“Oral temperatures between 98.7° and 100° F (37.1° to 37.8°C) are low-grade fevers.”	“These temperatures are normal. The body's normal temperature changes throughout the day. It peaks in the late afternoon and evening. **A true low-grade fever is 100°F to 102°F (37.8°–39°C).”**	Body temperatures vary by age, sex, daytime, physical activity, etc. Fever ≥39°C may precipitate inconsequential seizures in <5% of children.
	**“SUMMARY. Keep in mind that fever is fighting off your child's infection. Fever is one of the good guys.”** [PYREXIA IS ESSENTIALLY A BRAIN-CONTROLLED HARMLESS MECHANISM TO FIGHT OFF INFECTIONS FOR WHICH WE MAY NOT HAVE OTHER THERAPEUTIC OPTIONS AVAILABLE. An incremental increase in a fever over days is part of a normal immune response and should not be reduced unless causing ‘discomfort'. Usually, fever little over 39 ^o^C clears most common infections, whereas temperature > 39 ^o^C may be needed for other pathogens requiring higher restrictive temperatures including the novel ones, *e.g.*, strains of influenzae, measles, SARS-CoVs].		**Additionally, fevers at** • ~***39*°*C generates the heat shock response*** to prepare the host to deal with higher temperatures later. • **~*40*°*C enhances interferons production*** if viruses are still around (permissive) and prepares the host for a safer inflammation resolution. **Reduction of fever in diseases** with limited antibiotics/ antivirals availability **increases the chances of complications and mortality**

*Unless indicated otherwise “fevers” refers to the temperature increase caused by acute infection or ‘Pyrexia'. The information in quotes “…” is taken verbatim from the Seattle Children's hospital web page (updated as of 13 May 2021) (72)*.

*Other information is provided to further clarify the meaning*.

*The temperatures are indicative, not absolute, and are the closest approximate. It may be a little different for each individual*.

### Adverse Effects of Antipyresis on the Body's Response to Infection

Suppression of initial low-grade fever that prepares host to minimize the damage from a future surge of cytokines besides keeping the infection down, would logically increase the risk of being exposed to sudden overwhelming cytokine storm and experience adverse complications due to host bodies' non-preparedness, i.e., reduced capacity to resolve the ensuing damaging inflammation (4–7, 13, 14, 58). The induced normothermia blunts the host's HSR and essentially its capacity to suitably respond to resolve inflammation triggered in any infection. Fever-induced HSR response through HSF1 controls the expression of proinflammatory cytokines, such as IL-6-one of the purported culprits behind cytokine storm and increased mortality in diseases precipitating acute respiratory distress syndromes (ARDSs) (1, 55, 56, 108–110).

The intact HSR circuitry along with HSF-1 that may be variously deficient in different backgrounds is required for appropriate cytokine response generation, protection from inflammation (55–57), and IgG response (111). Strikingly, most conditions currently associated with higher mortality in COVID-19 and other respiratory tract viral infections have increased prevalence of reduced and/or defective HSR, e.g., aging (112), diabetes (both types 1 and 2) (113, 114), ARDS (115), sepsis (116, 117), renal failure (118), cigarette smoking (119), chronic obstructive lung disease (120). Unnecessary antipyresis would be more problematic for these sections of the population.

The induced normothermia is known to increase viral shedding and prolong recovery (121, 122), increase mortality from pneumonia (123–126), and reduce the efficacy of antibiotics (73, 74). The individuals unable to generate appropriate HSR would be prone to adverse outcomes from unnecessary antipyresis. Meta-analysis of animal studies had indicated an increased pooled odds ratio (*OR*) of 1.34 for influenza mortality with the antipyretics use (127), whereas hyperthermia preconditioning indicated enhanced survival in various relevant conditions, such as sepsis (128), stroke (129), myocardial infarction (130), and hepatic ischemia (131).

### Divergence of Emergent Fever Management Guidelines

The current WHO guidelines for fever management explicitly stipulate that febrile illness of ≥39°C (≥102.2°F) presented with defined acute complications or discomfort could be considered for fever reduction (32). Furthermore, the treatment should focus on the cause of the fever rather than the fever itself. Refer to Table 2A for an excerpt from guideline (32, Chapter 10: Management of Fever; p.305) that restricts antipyresis to “children uncomfortable or distressed because of fever” and mentions it may not benefit otherwise active and alert children besides compromising the immune defense. Additionally, the fever reduction guidelines are part of “Supportive Care” (32) for “the most vulnerable”, *i.e*., ~5% of children <5-year-old showing complications/discomfort and genetically predisposed to febrile seizures/convulsions. The fever-reduction consideration should weigh the potential benefit being derived, as the commonly observed frightening seizures/convulsions are inconsequential to children's wellbeing (23, 32, 70, 72). However, measures must be taken to reduce the hazard of accidental choking, self-injury, etc., during febrile seizures in such predisposed children.

**Table 2A:** Fever reduction guidelines: World Health Organization (WHO), 2013.

**Fever management guideline**	**Comments**
**WHO GUIDELINES, 2013 (in “quotes”)** **(****32****)** “***10.3 Management of fever*** The temperatures given in these guidelines are rectal temperatures, unless otherwise stated. Oral and axillary temperatures are lower by approximately 0.5 °C and 0.8 °C, respectively.	• The section deals with both *pyrexia* (**brain-regulated during infections)** and *thermia* (unregulated, *e.g.*, heat shock) • In pyrexia, the body temperature is increased in response to the pathogen **to prepare to fight off an infection - including incapacitating the pathogen at the restrictive temperature**. Temperatures can rarely increase up to 42^o^C if the risk persists.
	• **FEVER IS GOOD FOR THE IMMUNE SYSTEM**.
**FEVER is** NOT ***an indication for antibiotic treatment*** **and MAY HELP THE IMMUNE DEFENSE AGAINST INFECTION.** High fever (> 39 °C or > 102.2 °F) can have harmful effects, such as: • reducing the appetite • making the child irritable • precipitating convulsions in some children aged 6 months to 5 years • increasing oxygen consumption (e.g. in a child with very severe pneumonia, heart failure or meningitis).	• **Unregulated sudden increase in temperature (thermia)** > **39** **°C** (103-105F) when the body is not prepared for the shock, **can be lethal** and **subject to emergency fever reduction**, WHEREAS ***Hyperpyrexia up to 108F** can be **well tolerated** and **remains an important mechanism for pathogen control*** (see **Table 1**. for facts and myths about pyrexia) • *Convulsions* occur in *genetically predisposed* children (<5%) but are essentially harmless (23).
**All children with fever should be examined for signs and symptoms that indicate the underlying cause of the fever, and should be treated accordingly (see Chapter 6, p. 149)**. ***Antipyretic treatment*** ***Paracetamol*** Treatment with oral paracetamol should be restricted to children aged ≥ 2 months who have a fever of ≥ 39 °C (≥ 102.2 °F) AND are uncomfortable or distressed because of the high fever. CHILDREN WHO ARE ALERT AND ACTIVE ARE UNLIKELY TO BENEFIT from paracetamol.”	***Asks for the treatment of the ‘cause'. Chapter 6, p. 149 deals with antibiotic choice**,* making the guidelines more aligned to bacterial diseases. ***At higher temperatures, antibiotics efficiency increases, so antibiotics administration without antipyretics would be more efficient***. • The treatment of ≥ 39 °C fever should only be considered when it is ‘uncomfortable' or ‘causing distress' in children. • Antibiotics coadministration with antipyretics for most of the common bacterial diseases would not affect disease resolution. However, **REDUCING FEVER in VIRAL DISEASES with causative agents having** **≥39**^**o**^**C restrictive temperature**, e.g., strains of Influenzae, Measles, SARS-CoVs, **the effects can vary from inconsequential to severe (death)** depending upon the presence of preexisting immunity, previous heat shock response/temperature and ability to handle cytokine storm.

It may be opportune to upwardly revise the temperature range for antipyresis consideration. Soon after the publication of the first guideline by the WHO in 2000 (132), a meta-analysis published in the bulletin of the WHO (70) identified 41°C as “normal febrile range,” highlighted the continued practice of antipyresis as “parents and health professionals routinely treat fever in young children” despite the clear-cut realizations of “fever helps survival during infection, and that antipyresis increases mortality” in many diseases and the “potential for hepatotoxicity” and “overdosage” (70, 89–92). Moreover, it indicates “the WHO recommendations for the management of fever in children include the use of paracetamol for children with fever of ≥39°C” despite “insufficient data, however, support this recommendation” and suggest “We recommend that health professionals should not be encouraged to give antipyretics routinely to febrile children. Treatment should only be given to those children in obvious discomfort or those with known painful conditions” (70). The revised fever reduction guidelines were published by the WHO in 2013 to increase awareness among healthcare providers and parents, and to improve the adherence to appropriate practices (32) (as shown in Table 2A). However, the rational use of antipyresis may have further decreased during the COVID-19 pandemic with the publication of confusing and contradicting guidelines by different national agencies suggesting anything from 37.8 to 39°C fit for antipyresis (26–31). A few illustrative guideline examples are given in Table 2B.

**Table 2B:** Fever reduction guidelines: others during COVID-19 pandemic.

**Fever management guidelines**	**Comments**
**NATIONAL HEALTH SERVICE. FEVER IN ADULTS. 2020 (26)**. **The page includes even reference to COVID-19 management, states “*****Fever helps your body fight infections by stimulating your immune system: your body's natural defense. By increasing your body's temperature, a fever makes it harder for the bacteria and viruses that cause infections to survive***.**”** **BUT** **considers “*****fever/high temperature (37.8°C or greater)*****” and goes on to suggest it to be “*****fit for treatment”*** **on the unqualified** ***“uncomfortable feelings associated with a fever”***.	• **It contradicts WHO guidelines for fever reduction** in infectious diseases, which specifically ask for cause treatment > 39 °C, not just any fever in any infection that too just > 37.8 °C. • **The “uncomfortable feeling” in the guidelines alluding to the explicit definition of ‘uncomfortable' needing treatment in WHO guidelines (2013) for children that are known to physicians could create confusion in the general public promoting unnecessary medication**. • **The unqualified ‘uncomfortable feelings' are open to conjecture by the public when considering what is fit for treatment**.
**The supposed guidance given later for adults leaves much to individuals' interpretation whether to take antipyretics or not, i.e.**, **“Treating a fever** **Most fevers will improve of their own accord in a few days. However, there are a number of things you can do to help the uncomfortable feelings associated with a fever**. 1. Don't over dress… 2. Drink more fluids, …	
3. Take a medicine that reduces fever such as paracetamol (unless you're allergic or have been told by a healthcare professional that you can't take it).”	In the limited access of well-qualified practitioners during the pandemic situation, antipyretics usage for any fever could essentially contribute to increased hospital visits, admissions, complications, and mortality by unspecified numbers.
**The link provided for ‘ibuprofen' use for those who may like to use it instead of paracetamol is even more confusing, i.e.**, **“Ibuprofen** **(****28****)**	
*There's no evidence to show a link between ibuprofen, or other non-steroidal anti-inflammatory medications (NSAIDs), and catching or making coronavirus worse*. Paracetamol or ibuprofen can be used to help with the symptoms of coronavirus if needed, unless your doctor has told you paracetamol or NSAIDs are not suitable for you…”	The ‘**No evidence to show a link**' or the ‘Lack of evidence' is **NOT** equivalent to the ‘**Evidence of no link**' or the ‘Evidence of no effect'. The **latter should be relied upon in practice**, not the former. ***Usually in situations of ‘Lack of Evidence', the ‘theoretical considerations prevail'***.
**The ‘Coronavirus (COVID-19): Self-care advice' page goes on to advice as under:** **“Treating a fever at home** **It's safe to treat most fevers at home. However, you may be at risk of becoming dehydrated**.	
Do	**Antipyretics alone are known to prolong recovery and increase transmission of respiratory tract infections** especially for microbes with no therapeutic agent available. (see main text for examples) **STICK TO WHO GUIDELINES, 2013 (32)**
✓ wear loose, comfortable clothing… ✓ drink more fluids … ✓ monitor your pee color … ✓ take paracetamol if you have a temperature – always follow the manufacturer's instructions…”	
**INDIAN COUNCIL OF MEDICAL RESEARCH, INDIA (34)**. **For acute fever management, it states: “Management of Acute Fever. Chapter 2** **2.1.7 Principles of empiric therapy**	
**a. Supportive: Acetaminophen 650 mg every 6 hours round the clock is advisable, accompanied by tepid sponging for fever** **>103 F. Replace fluid and electrolytes as required**.	It considers >103 F as acute illness and recommends giving antipyretics, even acknowledging antibiotics may be of no use.
**b. No antibiotics are required for the majority of patients with acute febrile illness without an obvious clinical diagnosis.”**	
**The FAQ for COVID-19** **(****31****)** **states:**	It defeats the purpose of WHO's recommendations where the cause is to be treated, not the fever *per se*. **STICK TO WHO GUIDELINES, 2013 (32)**
**“What can I take (for) pain or fever?** • **…Paracetamol is one of the safest pain killers to use if needed.”**	
**CENTERS FOR DISEASE CONTROL AND PREVENTION (CDC), USA (27)**. **“CDC considers a person to have a fever when he or she has a measured temperature of 100.4°F (38°C) or greater, or feels warm to the touch, or gives a history of feeling feverish”**	**The CDC at multiple places clarifies that it considers** 100.4° F (38° C) or above a fever for diseases. **STICK TO WHO GUIDELINES, 2013 (32)**
**NATIONAL INSTITUTES OF HEALTH (NIH), USA (29)**	
**COVID-19 treatment guidelines (Last Updated: April 21, 2021) do not specifically elaborate on fever management. However, ‘Outpatient Management of Acute COVID-19' under ‘Symptom Management' p. 41 rather cursorily advises:** **“Symptomatic treatment includes using over-the-counter antipyretics, analgesics, and antitussives for fever, headache, myalgias, and cough.”**	**May be appropriate for promoting people to seek medical advice, but cursory remarks could promote inappropriate intake**. The widespread availability and use of antipyretics can increase complications and mortality from seasonal viral diseases including COVID-19. **Specific recommendations cautioning against antipyresis would have been better as the risk of overuse had increased during the COVID-19 pandemic**. **STICK TO WHO GUIDELINES, 2013 (32)**

*The guidelines floating around for COVID-19 are for the use of qualified physicians. These guidelines would need to go the extra mile to be in the public domain. These guidelines need to be aligned with WHO guidelines on fever management. Extra caution is desired when in the public domain, as any unqualified remark open to alternative interpretation may cause more harm than good to public health. It would have the potential to result in health issues, extra hospital visits, complications, and deaths putting pressure on the already overstretched medical systems of most countries during the COVID-19 pandemic*.

### What More Could Be Done?

The existing guidelines for fever management in the public domain (26–31) should be clarified and made explicit in stating the dangers of unnecessary fever reduction and emphasizing the necessity of fever in resolving infectious diseases. Inclusion of explanatory myth dispelling statements suitable for the public could help allay the fear of pathogen-hyperpyrexia and increase the awareness about its positive benefits as demonstrated (72). The extant guidelines may be either made strictly for the guidance of medical practitioners or must explicitly state the meanings of “uncomfortable,” “fit for treatment,” and “fever that needs treatment.” The guidelines should consider stating the “danger signs” when emergency medical attention is desired and identify the small minorities who are at greater risk of complications from higher temperatures, e.g., pregnant women (3–4 weeks, ≥39°C), children <5-year-old, frail, and deficient HSR individuals (refer to Sections above).

Public awareness should be increased about the essential role of the routine gradual raising of fever up to 39–42°C in promoting various protective (self-preserving) and offensive (pathogen clearance) measures for an amicable resolution of acute infectious diseases, e.g., immunity stimulation; HSR and anti-oxidative system activation to protect tissues from ensuing inflammatory response; antiviral response activation and enhancement; pathogen growth restriction/killing and clearance through nutrient deprivation, increased chemotaxis, phagocytosis, and reactive radical formation. The 39–42°C fever is desirable in the resolution of infections caused by the novel or high temperature adapted pathogens (restrictive temperature: 40–42°C) with limited treatment options, e.g., SARS-CoV-2, strains of Influenzae, measles, and pneumococcus.

## CONCLUSION

The potential for fever mismanagement during the COVID-19 pandemic has further increased with the publication of numerous confusing guidelines. Unless corrective actions are taken immediately, it would further aggravate the situation causing increased morbidity and mortality from different infectious diseases including the common seasonal viral diseases and COVID-19. Unnecessary antipyresis could promote increased pathogen transmission rates, complications, hospitalization, and associated deaths. The awareness campaigns to dispel the myths and misconceptions surrounding fever, and promotion of evidence-based fever management practices should be undertaken for the public good.

## Data Availability Statement

The original contributions presented in the study are included in the article/supplementary material, further inquiries can be directed to the corresponding author.

## Author Contributions

SS conceptualized and wrote the manuscript. RS and DK contributed to critical inputs and wrote and refined the manuscript. All authors contributed to the article and approved the submitted version.

## Conflict of Interest

The authors declare that the research was conducted in the absence of any commercial or financial relationships that could be construed as a potential conflict of interest.

## Publisher's Note

All claims expressed in this article are solely those of the authors and do not necessarily represent those of their affiliated organizations, or those of the publisher, the editors and the reviewers. Any product that may be evaluated in this article, or claim that may be made by its manufacturer, is not guaranteed or endorsed by the publisher.
